# Agonist signalling properties of radiotracers used for imaging of dopamine D_2/3_ receptors

**DOI:** 10.1186/s13550-014-0053-3

**Published:** 2014-10-07

**Authors:** Jan-Peter van Wieringen, Martin C Michel, Henk M Janssen, Anton G Janssen, Philip H Elsinga, Jan Booij

**Affiliations:** 1 Department of Nuclear Medicine, Academic Medical Center, University of Amsterdam, Meibergdreef 9, Amsterdam, 1105, AZ, The Netherlands; 2 Department of Pharmacology, Johannes Gutenberg University, Mainz, Germany; 3 SyMO-Chem BV, Eindhoven, The Netherlands; 4 GE Healthcare, Eindhoven, The Netherlands; 5 Department of Nuclear Medicine and Molecular Imaging, University Medical Center Groningen, University of Groningen, Groningen, The Netherlands

**Keywords:** Dopamine D2/3 receptor, PET/SPECT, Intracellular signalling

## Abstract

**Background:**

Dopamine D_2/3_ receptor (D_2/3_R) agonist radiopharmaceuticals are considered superior to antagonists to detect dopamine release, e.g. induced by amphetamines. Agonists bind preferentially to the high-affinity state of the dopamine D_2_R, which has been proposed as the reason why agonists are more sensitive to detect dopamine release than antagonist radiopharmaceuticals, but this theory has been challenged. Interestingly, not all agonists similarly activate the classic cyclic adenosine mono phosphate (cAMP) and the ?-arrestin-2 pathway, some stimulate preferentially one of these pathways; a phenomenon called biased agonism. Because these pathways can be affected separately by pathologies or drugs (including dopamine releasers), it is important to know how agonist radiotracers act on these pathways. Therefore, we characterized the intracellular signalling of the well-known D_2/3_R agonist radiopharmaceuticals NPA and PHNO and of several novel D_2/3_R agonists.

**Methods:**

cAMP accumulation and ?-arrestin-2 recruitment were measured on cells expressing human D_2_R.

**Results:**

All tested agonists showed (almost) full agonism in both pathways.

**Conclusions:**

The tested D_2/3_R agonist radiopharmaceuticals did not exhibit biased agonism *in vitro*. Consequently, it is likely that drugs (including psychostimulants like amphetamines) and/or pathologies that influence the cAMP and/or the ?-arrestin-2 pathway may influence the binding of these radiopharmaceuticals.

## Background

The dopamine (DA) system plays a central role in several neuropsychiatric disorders including Parkinson's disease and schizophrenia. DA D_2_ receptor (D_2_R) antagonists are used to reduce psychotic symptoms, whereas D_2_R agonists are commonly used in the treatment of Parkinson's disease. Consequently, radiopharmaceuticals targeting D_2_Rs are of value to obtain insight in the pathophysiology of these brain disorders.

The D_2_R is a subfamily within the superfamily of G-protein-coupled receptors (GPCRs) and contains several subtypes including some in the D_2_-like subfamily, the D_2_ (splice variants D_2short_ (D_2_S) and D_2long_ (D_2_L) [1]), D_3_ and D_4_ receptors [2]_._ They primarily couple to the G_i/o_ type of G-proteins to inhibit the enzyme adenylyl cyclase in producing cyclic adenosine mono phosphate (cAMP) [2]. Like other GPCRs, they exhibit interconvertible high- and low-affinity states for agonists in vitro [3]. In the high-affinity state, the receptor is coupled to the G-protein and this is considered to be the active state of the receptor. In the low-affinity state, the receptor is uncoupled and consequently inactive.

Dopamine D_2/3_ receptor (D_2/3_R) agonist radiopharmaceuticals for positron emission tomography (PET) have been developed successfully (e.g. ^11^C-NPA (N-propylnorapomorphine) and ^11^C-PHNO ((+)-4-propyl-9-hydroxynaphthoxazine)), and such agonists are more sensitive in detecting DA release in humans compared to antagonists like ^11^C-raclopride [4]-[7]. It has been proposed that the reason for this increased sensitivity may be that D_2/3_R agonist radiopharmaceuticals bind preferentially to the high-affinity state of the dopamine D_2_R, whereas antagonists do not differentiate between the high- and low-affinity states [8]-[10]. However, this proposal depends upon the existence of two affinity states *in vivo* and the results of recent studies challenged this theory [11]-[13].

**Figure 1:**
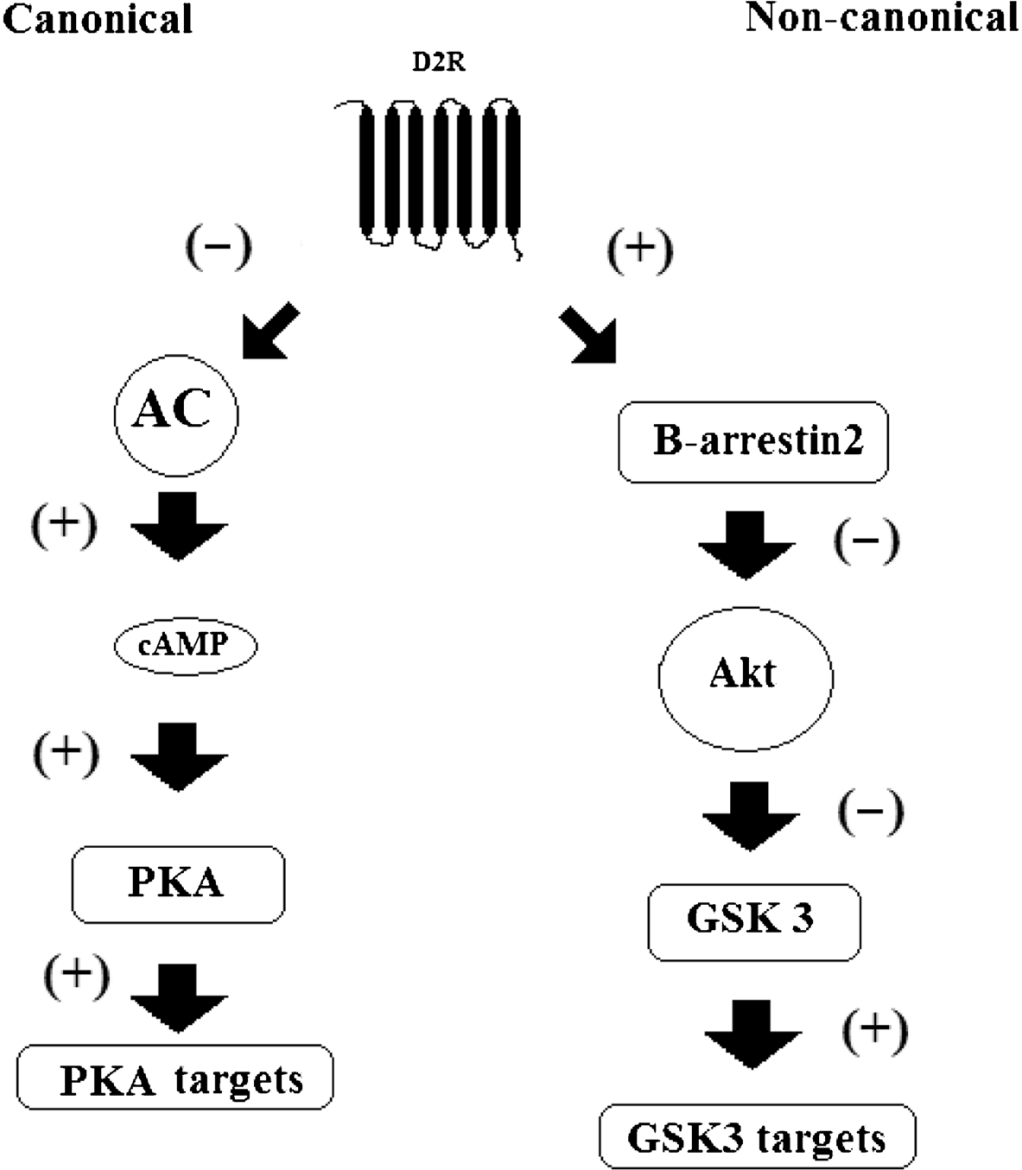
**Canonical (G-protein-dependent) and non-canonical (G-protein-independent) signalling of D2R.** Beta-arrestin-2 inactivates Akt resulting in increased activation of GSK3. D_2_R, dopamine D_2_ receptor; AC, adenylyl cyclase; cAMP, cyclic adenosine mono phosphate; PKA, protein kinase A; Akt, protein kinase B; GSK3, glycogen synthase kinase 3.

Originally, DA receptors (and other GPCRs) were thought to signal intracellularly only through their G-proteins. However, it was shown recently that besides this (canonical) pathway, DA receptors can also exert effects through proteins of which it was initially thought that they regulate receptor desensitization (non-canonical pathway, Figure 1). This cAMP-independent mechanism involves the adaptor protein ?-arrestin-2. Compounds can have distinct patterns of responses on these pathways; this phenomenon is called `biased agonism¿ or `ligand-directed signalling¿ [14],[15].

Activation of the ?-arrestin-2 pathway may play a role in the increased sensitivity to detect dopamine release *in vivo*. Activation of this pathway eventually leads to the regulation of glycogen synthase kinase 3 (GSK3), a protein that is involved in many DA-dependent behaviours [16]. Many drugs (antipsychotics, antidepressants, lithium) affect this cascade, and recently compounds, based on the aripiprazole scaffold, were discovered that are functionally selective for the ?-arrestin-2 pathway [17],[18]. Consequently, it is important to characterize pharmacologically the intracellular pathways that DA D_2/3_R agonist radiopharmaceuticals act on. This information is not only relevant when the radiopharmaceuticals are used to evaluate actions of novel drugs which may show biased agonism but also because ?-arrestin-2 itself may be involved in the desensitization of DA receptors and as such in the detection of DA. Literature about biased signalling of currently available agonist PET radiotracers ^11^C-NPA and ^11^C-PHNO at cloned DA receptors does not exist, and therefore, we tested unlabelled (`cold¿) NPA and PHNO for agonism in both the cAMP and ?-arrestin-2 assays.

Additionally, and in a broader perspective, we are developing new DA D_2/3_R agonist radiotracers for PET and single-photon emission computed tomography (SPECT) imaging, where the investigated compounds are based on the 2-aminomethylchromane (AMC) scaffold. We have tested agonism for our AMC compounds by means of a cAMP assay and have found that most of these compounds showed full agonism for this canonical pathway [19]. As for NPA and PHNO, also for these new AMC tracers, we were interested to evaluate if they would show biased agonism. Finally, the D_2/3_R antagonist SPECT tracer ^123^I-iodobenzamide (^123^I-IBZM) is used in many studies including studies on Parkinsonism [20] and on amphetamine-induced DA release [21]. Based on early studies in rats showing that IBZM was a potent inhibitor of an apomorphine-induced syndrome (apomorphine is a D_2_R agonist) of hyperactivity and stereotypy, it was concluded that IBZM was a DA antagonist [22]. As no other research examining the functional agonism or antagonism of IBZM has been reported, we also tested IBZM for biased agonism. Of note, studies with other receptors show that antagonists for one signalling pathway of a receptor may be agonists for another pathway.

Accordingly, in this study, we have tested if clinically used as well as recently developed D_2/3_R radiopharmaceuticals show biased agonism.

## Methods

**Figure 2:**
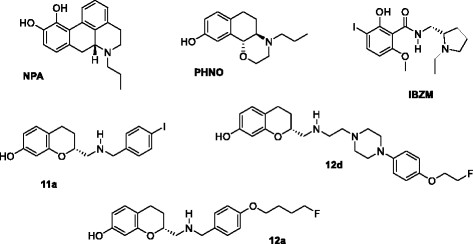
**The tested compounds.** The cold analogues of the clinically applied tracers NPA, PHNO and IBZM and from the developed series of AMC compounds 11a, 12a and 12d (published in [19]).

The agonists *R*(-)-propylnorapomorphine HCl, pergolide mesylate and DA HCl were obtained from Sigma-Aldrich (Zwijndrecht, The Netherlands). (+)PHNO HCl was obtained from Axon Medchem (Groningen, The Netherlands). The cold-labelled (i.e. iodine-127-labelled) antagonist iodo-6-methoxybenzamide (IBZM) was a kind gift of GE Healthcare (Eindhoven, The Netherlands). We have previously presented the synthesis and evaluation of a new group of cold-labelled D_2/3_R agonist tracers based on an AMC scaffold, including *(R)*N-[7-hydroxychroman-2-yl]-methyl 4-iodobenzyl amine (codenamed 11a), (*R*)-2-[(4-(4-fluorobutoxy)benzylamino)methyl] chroman-7-ol (codenamed 12a) and (*R*)-1-(4-(2-fluoroethoxy)phenyl)-4-(4-(7-hydroxychroman-2-yl)-3-azabutyl)-piperazine (codenamed 12d) [19] (see Figure 2).

### cAMP accumulation assay and receptor binding assay

As described in detail earlier [19], cAMP accumulation was measured in HEK 293 cells stably expressing the human DA D_2_L receptor with an assay from PerkinElmer (Waltham USA). Receptor binding experiments were done on the membranes obtained from the above mentioned cells using ^3^H-spiperone as radioligand.

### ?-arrestin-2 recruitment assay

The PathHunter¿ eXpress human DA receptor D_2_L CHO-K1 ?-arrestin-2 GPCR assay from DiscoveRx (Fremont, CA, USA) was used according to the manufacturer's protocol. Cells were seeded at a density of 8,000 cells/well in a 96-well plate in 100 ?l PathHunter¿ CP reagent and incubated 48 h in a humidified atmosphere at 5% CO_2_ at 37°C. After 48 h, the compounds dissolved in Hank's balanced salt solution supplemented with 5 mM HEPES and 1.1% DMSO were added to the wells in duplo. Then a detection mixture was made and added to the wells. The cells were incubated for 60 min at room temperature, and subsequently chemiluminescence was measured on a Wallac Victor (PerkinElmer, Zaventum, Belgium).

## Results

### cAMP

In our previous studies the novel AMC compounds 11a, 12a and 12d bound with a high-affinity to the DA D_2_-high receptor and were full agonists compared to DA itself; DA, 11a and 12a were similarly potent, and 12d was a little more potent [19]. NPA and PHNO were also full agonists, displaying E_max_ values even higher than those of DA that was set at 100% efficacy as a reference. Furthermore, NPA and PHNO were, respectively, 1 log and 2 log units more potent than DA. The SPECT tracer IBZM was an inverse agonist. All data are compiled in Table 1.

**Table 1:** Radioligand binding and functional activities of different compounds*

		**cAMP**		**?-arrestin-2**	
**Compound**	**pK**_**i**_**D**_**2**_**-high**	**Potency**	**Efficacy**	**Potency**	**Efficacy**
		pEC_50_	%	pEC_50_	%
Dopamine	6.97^¿^	9.22^¿^	100^¿^	6.97	100
NPA	10.13^¿^	10.31	118	9.67	103
PHNO	9.47^¿^	11.03	114	9.39	99
11a	8.69^¿^	9.16^¿^	86^¿^	8.26	81
12a	8.26^¿^	9.42^¿^	96^¿^	8.36	88
12d	8.30^¿^	9.82^¿^	94^¿^	8.61	88
IBZM	6.99^¿^	9.08	Inverse agonist	-	Neutral antagonist

*pKi, pEC_50_ and E_max_ values are the average of at least two duplicate experiments with standard deviations (SD) values that were less than a third of the mean.

^¿^Parts published earlier in van Wieringen et al, 2014 [19].

^¿^For IBZM no high-affinity state was detected; pK_i_ value represents affinity for the total number of D_2_ receptors.

### ?-arrestin-2

**Figure 3:**
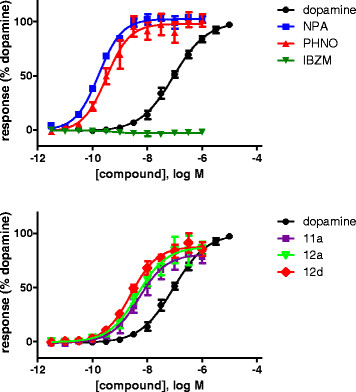
**Concentration response curves of different compounds for arrestin signalling.** Quantitative analysis of the data is shown in Table 1.

NPA, PHNO, 11a, 12a and 12d demonstrated full agonism for this pathway (using DA as the 100% reference), whereas IBZM was a neutral antagonist (Figure 3). In the ?-arrestin-2 pathway, all tested compounds had higher potencies than DA (pEC_50_ of 6.97) with NPA being the most potent (pEC_50_ of 9.67). See Figure 3 and Table 1 for all data.

The potencies of all tested compounds, as measured in the two functional tests, correlated (*r* =0.80; Spearman's rank correlation).

## Discussion

We found that all agonist compounds tested that were active in the cAMP assays were also active in ?-arrestin-2 recruitment with an efficacy similar to that of DA. Moreover, the potencies of all agonists in the two functional assays were well correlated with a slope close to unity (1.046?±?0.3965). However, their potency was consistently higher in the cAMP than in the ?-arrestin-2 assay, especially for DA and PHNO. This may be explained by the presence of a receptor reserve in the cells used in the cAMP experiments causing signal amplification while the arrestin recruitment assay measures a more 1:1 interaction of the receptor to arrestin. All in all, the present data do not support biased agonism for these compounds.

Skinbjerg and colleagues recently demonstrated that the striatal binding potential (BP_ND_) of the agonist ^11^C-MNPA and the antagonist (or inverse agonist; for discussion see below) ^18^F-fallypride was not different in wild-type mice and ?-arrestin-2 knockout mice [23]. The results of this study suggest that a modified arrestin pathway does not affect radiotracer binding during `baseline¿ conditions, independent of agonist signalling properties of the radiotracer. A modification in the arrestin pathway is more likely to be demonstrated using a pharmacological challenge, as shown in the amphetamine studies by Skinbjerg and co-workers [23]. The current work, therefore, appears most relevant to drug interaction studies, and consequently may shed new light on the reason why agonist D_2/3_R radiopharmaceuticals are more sensitive to detect DA release than antagonists [4]-[7]. More specifically, it has been demonstrated that arrestin desensitizes the D_2_R and prepares it for internalization by hindering the coupling of the G-protein to the receptor. Consequently, the desensitized receptor is in the low-affinity state [24]. So, after agonist binding, the receptor is switched to the low-affinity state and kept in that state by arrestin; thus, theoretically the density of D_2_ receptors in the high-affinity state decreases after binding with an agonist that recruits arrestin.

DA release is measured with PET or SPECT by assessing the difference in binding potential for radiotracers that label DA D_2/3_Rs after a dopaminergic challenge as compared to a baseline condition [8],[21],[25]. DA can displace the radiotracer from the receptor (competition model), but the lower binding potential after a DA challenge may also be explained partly by internalization of DA D_2/3_Rs. In a typical PET/SPECT study, DA release is measured 1 h after a DA challenge [8],[21], and internalization of DA D_2/3_Rs has been reported in a ^11^C-raclopride PET study in cats already 1 h after the administration of the DA releaser amphetamine [26]. Moreover, Guo and co-workers [27] demonstrated a lower binding affinity of agonist and antagonists radiotracers for internalized DA D_2/3_Rs, possibly reflecting a poorer accessibility than to surface receptors. Since, the ?-arrestin pathway plays an important role in the internalization of DA D_2/3_Rs, it is important to know if a radiotracer is biased towards or away from ?-arrestin translocation. Radiotracers that are biased towards cAMP may not promote ?-arrestin-mediated receptor internalization and in theory may be more accurate tools to detect DA release. Recently cAMP-specific compounds have been developed for the dopamine D_2_R and may offer the opportunity to be developed further to radioligands in future investigations [28]. These propositions may have implications for the interpretation of results from clinical studies with agonist tracers, particularly pharmacological challenge studies, e.g. with DA-releasing psychostimulants.

Since all tested agonists activate the cAMP as well as the ?-arrestin-2 pathway, it is likely that they will also activate both GSK3 (via ?-arrestin-2) and protein kinase A (via cAMP). This information is of importance when these radiopharmaceuticals are used to study or evaluate (novel) drugs. Dopaminergic drugs, like antipsychotics, but also drugs affecting other monoamine systems like the serotonin releaser fenfluramine, exert actions on the ?-arrestin-2 pathway. Lithium, a drug used in the treatment of bipolar disorder, was found to have a direct inhibiting action on GSK3 [16]. Finally, compounds based on the aripiprazole scaffold were recently discovered that are functionally selective for the ?-arrestin-2 pathway and that show robust antipsychotic effects in rodents [17],[18]. So, it is likely that drugs that influence the canonical and/or the non-canonical pathway, may influence indirectly the *in vivo* binding of our presently tested D_2/3_R agonists, including the radiotracers ^11^C-NPA and ^11^C-PHNO, which are frequently used in human studies [5],[7].

Our novel finding that IBZM showed inverse agonism in the cAMP assay is in line with recent findings that the antipsychotics nemonapride and sulpiride (similar to IBZM, benzamides), and also antipsychotics from other chemical classes (e.g. butyrophenones like haloperidol), that were initially believed to be DA receptor neutral antagonists are now re-classified as inverse agonists by the results of studies using functional assays [29]. It is alleged that inverse agonists bind preferentially to the inactive state of the receptor [30], so this might indicate that *in vivo*^123^I-IBZM predominantly binds to striatal D_2_Rs in their inactive low-affinity state. Since ^11^C-raclopride PET and ^18^F-fallypride are frequently used to assess DA release *in vivo*[5],[7],[23], it may be of relevance to test whether raclopride and fallypride are also inverse agonists for cAMP instead of antagonists. If our proposition is true that benzamide radiopharmaceuticals *in vivo* bind predominantly to DA D_2/3_Rs in their low-affinity state, this will extend the discussion on why benzamides tracers are less sensitive than agonists D_2/3_R radiopharmaceuticals (like ^11^C-PHNO) to detect DA release *in vivo*[5],[7].

Nevertheless, more functional pharmacological characterization studies on agonist and antagonist (or inverse agonists) D_2/3_R radiopharmaceuticals are needed to fully understand the mechanism how dopamine release can be detected *in vivo* using such radiotracers.

In this study, we focused on the D_2_R. Since all developed radiopharmaceuticals to image this receptor also bind to the D_3_R, it may be of interest to evaluate in future studies also how these radiotracers act on intracellular signalling of the D_3_R.

## Conclusions

The agonist compounds tested exhibited agonism for both the cAMP and the ?-arrestin-2 pathways, and no evidence was found for biased agonism. This information is crucial for the interpretation of findings with these tracers, especially in drug interaction studies. In addition, our data demonstrate, for the first time, that the benzamide IBZM is an antagonist for both ?-arrestin-2 recruitment and cAMP formation, displaying inverse agonist properties for the latter.

## Competing interests

MCM is an employee of Boehringer Ingelheim, a company marketing the DA D_2_ agonist pramipexole. AGMJ is an employee of GE Healthcare, a company marketing the DA D_2/3_ radiopharmaceutical ^123^I-IBZM. JB is a consultant at GE Healthcare. The other authors declare that they have no competing interest.

## Authors¿ contributions

JPvW carried out the molecular signalling experiments, analysed the data, interpreted the results and drafted the manuscript. MCM was responsible for the design of the cAMP experiments, helped in analysing the data, interpreting the results and critically revising the manuscript. HMJ synthesized the aminomethylchroman molecules, helped in interpreting the results and in critically revising the manuscript. AGMJ helped interpreting the data and in critically revising the manuscript. PHE helped interpreting the data and in critically revising the manuscript. JB helped in the design of experiments, the interpretation of the data and in drafting the manuscript. All authors read and approved the final manuscript and agreed to be accountable for all aspects of the work in ensuring that questions related to the accuracy or integrity of any part of the work are appropriately investigated and resolved.
